# The Myofunctional Rehabilitation of Atypical Swallowing in Children and Adolescents With Stress‐Related Detrimental Habits: Single‐Center Longitudinal Study in Ukraine

**DOI:** 10.1002/cre2.70376

**Published:** 2026-05-20

**Authors:** Nataliia Makhlynets, Myroslava Kokoshko, Nataliia Kozan, Zinovii Ozhogan, Taras Kotyk

**Affiliations:** ^1^ Ivano‐Frankivsk National Medical University Ivano‐Frankivsk Ukraine; ^2^ Taras Shevchenko National University of Kyiv Kyiv Ukraine

**Keywords:** adolescent, anxiety, child, deglutition, habits

## Abstract

**Objectives:**

War‐related stress may exacerbate parafunctional oral/postural habits and hinder the normalization of swallowing in children. Evidence for low‐burden myofunctional protocols remains limited, particularly in juveniles living in high‐stressor settings. This study described short‐term trajectories of swallowing rehabilitation status in Ukrainian orthodontic patients treated with a standardized FroggyMouth‐based program during the wartime period.

**Material and Methods:**

This study used a single‐center longitudinal observational design (March 2022–June 2024) with consecutive enrollment. In total, 120 orthodontic patients aged 6–17 years with atypical swallowing and oral/postural habits were prescribed FroggyMouth (15 min/day during a quiet activity). Reassessment was performed at ~5 (T1) and ~10 weeks (T2). Rehabilitation status (RS) was classified as RS0 (baseline), RS1 (engrammation), and RS2 (automation). Paired change in the three‐level RS distribution between T1 and T2 was assessed. Habit profiles and anxiety were recorded (age‐stratified STAI‐C/STAI‐Y) and summarized descriptively.

**Results:**

Oral/postural habits were frequently observed: resting the head on the hand in 80% of patients; sleeping on the hand in 36.7%; watching TV with the mouth open in 25.0%; and mouth breathing in 41.7%. Non‐nutritive sucking behaviors were uncommon. Anxiety varied across groups (means ~43–44 at ages 6–11 vs. ~50–55 at ages 12–17). RS distribution shifted toward later stages from T1 to T2 (RS0/RS1/RS2: 54.2%/25.8%/20.0% at T1 vs. 14.2%/22.5%/63.3% at T2). Age groups differed in the magnitude of within‐subject RS change (*p* < 0.001), while RS2 proportions at T2 were broadly similar.

**Conclusions:**

In this uncontrolled clinic‐based cohort, a standardized FroggyMouth‐based program demonstrated a short‐term shift toward RS2 between 5 and 10 weeks, reaching 63.3% (95% CI 54.1–71.9) overall at 10 weeks. These observational data quantify short‐term change; longer follow‐up and comparative designs are needed to establish persistence and relative effectiveness.

## 1 Introduction

Since February 2022, a substantial proportion of Ukrainian families has been living under chronic stress related to war exposure, displacement, recurrent air alerts, disrupted schooling, and persistent uncertainty. Children, similarly to adults, suffer from direct and indirect war effects, leading to the development of clinically relevant posttraumatic stress reactions (Martsenkovskyi et al. 2024).

Measurable deterioration of the child's mental health during the war period was also observed from the parents' perspective (McElroy et al. 2024). On the other hand, stress and anxiety potentially are linked with maladaptive behaviors observed among orthodontic patients (Silva et al. 2021).

Orofacial functions (breathing, swallowing, and tongue–lip posture at rest) are linked to dentofacial development and may affect the stability of occlusal correction. Atypical swallowing is one among such clinically relevant functional patterns. Usually, the transition toward a mature swallowing pattern occurs in early childhood, but atypical patterns may persist and become clinically relevant in mixed and permanent dentition stages (Scribante et al. 2024). In addition, atypical swallowing is often associated with mouth breathing (Gómez‐González et al. 2024), leading to synergic impact on the development of dentofacial anomalies (Lin et al. 2022; Barata et al. 2021). Thus, screening of orthodontic patients for atypical swallowing with further correction is crucial for harmonious growth of the dentofacial region (Scribante et al. 2024).

Oral and postural habits (e.g., digit sucking and related non‐nutritive sucking behaviors) are reinforced through repetition and may be shaped by broader psychosocial context (Silva et al. 2021), simultaneously serving as adaptive mechanisms (Ilana 2020). Over time, habits become ingrained behaviors due to frequent repetition and possess a strong neurobiological framework (Kandel 2009; Smith and Graybiel 2016). On the other hand, detrimental oral habits have been consistently associated with malocclusion development and occlusal changes in early life, with the risk depending on the duration and persistence of the habit (Silva et al. 2021; Doğramacı and Rossi‐Fedele 2016).

Myofunctional rehabilitation is commonly used to correct atypical swallowing and related functional patterns in orthodontic patients. However, routine implementation is often limited by program duration, the need for frequent practice, and variable adherence—constraints that are especially relevant in children with anxiety and multiple stressors. Therefore, standardized low‐burden protocols are of particular practical interest. FroggyMouth has been described as a time‐limited daily protocol using a ready‐made appliance, intended to facilitate the acquisition of a more automatic swallowing pattern (Di Vecchio et al. 2019). While the available evidence base is limited and still evolving (Scribante et al. 2024; Di Vecchio et al. 2019; Fellus 2018), in Ukrainian settings, these devices' advantages make them a pragmatic option in routine orthodontic workflows where intensive therapy schedules may be unrealistic.

Within this context, there is limited data about short‐term trajectories of atypical swallowing rehabilitation in children and adolescents with pronounced stressors and anxiety under war‐related conditions. The present study aimed to assess short‐term changes in swallowing patterns in children and adolescents with oral/postural habits following a standardized Froggy Mouth‐based myofunctional rehabilitation program.

## 2 Materials and Methods

### 2.1 Study Design

Single‐center longitudinal observational study conducted at the Center of Dentistry of the University Clinic of Ivano‐Frankivsk National Medical University (Ivano‐Frankivsk, Ukraine) from March 2022 to June 2024.

The research was conducted in accordance with the Declaration of Helsinki; the study protocol was approved by the Ethics Committee of Ivano‐Frankivsk National Medical University (142/24, dated February 22, 2024). Written and verbal informed consent was obtained from all children's parents before participating in the study.

### 2.2 Participants and Eligibility Criteria

Consecutive patients aged 6–17 years who attended an orthodontic consultation because of dentoalveolar anomalies or tooth position changes and had clinically confirmed atypical swallowing at baseline were enrolled in the study. Patients did not present primarily for functional complaints; functional assessment was performed as part of the orthodontic examination.

Inclusion criteria: 6–17 years old, atypical swallowing, presence of oral and/or postural habits.

Exclusion criteria were defined to ensure the homogeneity of the research cohort and to minimize confounding variables (primary motor deficits or congenital structural defects) that could independently distort the assessment of functional patterns and their relationship to psychological stressors (Gyra et al. 2025; Zhao et al. 2021): craniofacial syndromes/significant craniofacial anomalies, a documented neurological or psychiatric condition likely to affect orofacial function or cooperation, or a history of traumatic brain injury/craniofacial trauma.

### 2.3 Participants Groups

Participants were stratified a priori into four age groups to reflect clinically distinct stages of dentition transition and skeletal maturation: 6–8 years (early mixed dentition), 9–11 years (late mixed dentition), 12–14 years (early permanent dentition and typical window of pubertal growth peak), and 15–17 years (late adolescence/post‐peak maturation) (Dinu et al. 2025; Dias and Gleiser 2009; Naderi et al. 2022; American Academy of Pediatric Dentistry 2025).

### 2.4 Data Sources and Measurements

Data collection included recording age, sex, presence of oral (lip biting, tongue thrusting, thumb/pencil sucking, mouth breathing), postural habits (resting hand on head, sleeping on hand, watching TV with open mouth), anxiety assessment, and stressors (free‐text response, e.g., war‐related factors, school, displacement). Clinical assessment included a swallowing assessment, performed jointly by two dentists, co‐authors, and recorded by consensus. Before enrollment, both evaluators reviewed the assessment criteria and conducted a brief calibration on pilot cases; formal intra‐ and inter‐examiner reliability was not quantified.

#### 2.4.1 Swallowing Assessment

Considering saliva swallowing is performed 1200–3000 times per day, its impact on dentofacial morphology is potentially more substantial compared to water/bolus swallowing (Jalaly et al. 2009). Swallowing was assessed clinically according to (Fellus 2020). The examiner placed the little finger against the inner surface of the cheek and asked the child to swallow saliva; labiomental (labial‐chin) contraction and buccinator/jugal pressure (force) on the finger were recorded. Atypical swallowing was diagnosed when swallowing showed labiomental contraction and/or increased buccinator/jugal pressure, consistent with persistence of a sucking‐swallowing pattern beyond the onset of mastication (Fellus 2020).

#### 2.4.2 Anxiety Assessment (STAI)

State Anxiety scale of the State‐Trait Anxiety Inventory for children (max score = 60) was used in 6 to 8‐ and 9 to 11‐year cohorts (Spielberger et al. 1973); State Anxiety scale of STAI (STAI Form Y‐1, max score = 80) (Spielberger et al. 1983) was restricted to adolescents aged 12–17 years (Shain et al. 2020). The higher values correspond to greater state anxiety.

### 2.5 Intervention: Myofunctional Rehabilitation

Patients with atypical swallowing entered a standardized myofunctional rehabilitation program using FroggyMouth. Use of FroggyMouth was selected because it provides a standardized, low‐burden regimen, designed to facilitate acquisition of a more automatic swallowing pattern, and is prescribed for short daytime use (e.g., while watching TV) (Scribante et al. 2024; Di Vecchio et al. 2019; Fellus 2015). Device size was selected and the intervention delivered according to the manufacturer's protocol; the prescribed use was 15 min daily during a quiet activity (e.g., TV, videogames) (Scribante et al. 2024; Di Vecchio et al. 2019; Fellus 2015; Froggy & Co. n.d.). Adherence was assessed at follow‐up by parent/guardian report.

Rehabilitation status (RS) was classified per the manufacturer's protocol as: RS0—baseline (pre‐treatment); RS1—engrammation (assessed at the intermediate visit, week 5); RS2—automation (evaluated at week 10), defined as corrected tongue posture at rest and during swallowing (Froggy & Co.).

The clinical schedule comprised baseline device fitting and follow‐up visits at ~5 and ~10 weeks. After 5 weeks of device use (T1), the RS status was assessed, and if RS1 was not reached, the “Tongue wrestling exercise” was performed in‐clinic with the following prescription: wear the device until the next visit (15 min/daily). In case of reaching RS1, patients were introduced to exercises—“Automating swallowing through olfaction” (prescribed 2–3 times per week till next visit) and “Automating through articulation” (prescribed to be repeated regularly at home until the subsequent evaluation if the in‐clinic counting test (counting aloud) showed tongue interposition—tongue visible between the dental arches) (Fellus 2020; Froggy & Co.). After RS2 is achieved, gradual weaning over 9–12 months was prescribed (Froggy & Co.).

### 2.6 Statistical Methods

The statistical analysis was performed in R v.4.5 (R Foundation for Statistical Computing, Vienna, Austria, 2025). Categorical variables will be reported as *n* (%); continuous variables as mean ± SD. RS was analyzed as an ordinal three‐level outcome (0/1/2) at T1 (5 weeks) and T2 (10 weeks). Within‐subject change in RS distribution between T1 and T2 was assessed by the Stuart–Maxwell test. The magnitude of individual change was expressed as Δ = RS(T2) − RS(T1) as Δ0/Δ1/Δ2 for each RS level outcome; between‐age differences in Δ distribution were assessed using an exact Fisher–Freeman–Halton test. The proportion of patients reaching RS2 (automation) at T2 was reported with an exact 95% CI. Power analysis was performed with the R‐*pwr* package v. 1.3. The *p*‐values < 0.05 were considered statistically significant.

## 3 Results

### 3.1 Participants and Follow‐Up

A total of 120 patients with clinically confirmed atypical swallowing were included (Table 1). Baseline oral and postural habits were frequently recorded, with postural patterns predominating. “Resting head on hand” was the most prevalent habit, while “sleeping on hand” was common, particularly among younger groups (up to 67%). “Watching TV with an open mouth” was most common among the 9–11‐year‐olds and was occasional in other age groups. Lip biting was most frequent in the 6–8‐year age group (36.1%) and was uncommon thereafter. Tongue thrusting and thumb/pencil sucking were rare. Mouth breathing was commonly observed in 9‐ to 14‐year‐old patients (up to 61% overall). Anxiety scores exhibited a pronounced age‐dependent upward trend. Furthermore, all study participants reported exposure to a variety of war‐related stressors. These included disruptions to formal schooling, impediments to peer socialization, and forced displacement. Participants also highlighted the psychological strain of frequent air raid alerts necessitating relocation to bomb shelters, alongside pervasive anxieties regarding potential homelessness, familial separation, the loss of a safe space, and exposure to distressing media content.

**Table 1:** Basic participants' characteristics.

Variable	6–8 years (*n* = 34)	9–11 years (*n* = 27)	12–14 years (*n* = 37)	15–17 years (*n* = 22)	Total (*n* = 120)
Age	6.3 ± 0.5	10.0 ± 0.6	12.7 ± 0.7	16.2 ± 0.7	10.9 ± 3.6
Sex (male/female)	14/20	9/18	18/19	11/11	52/68
Anxiety	44.5 ± 5.3^a^	43.6 ± 6.7^a^	49.7 ± 5.5^b^	55.2 ± 7.2^b^	
Resting head on hand, *n* (%)	25 (73.5%)	27 (100.0%)	25 (67.6%)	19 (86.4%)	96 (80.0%)
Sleeping on hand, *n* (%)	22 (64.7%)	10 (37.0%)	11 (29.7%)	1 (4.5%)	44 (36.7%)
Watching TV with an open mouth, *n* (%)	0 (0.0%)	19 (70.4%)	9 (24.3%)	2 (9.1%)	30 (25.0%)
Lip biting, *n* (%)	11 (32.4%)	6 (22.2%)	2 (5.4%)	0 (0.0%)	19 (15.8%)
Tongue thrusting, *n* (%)	0 (0.0%)	2 (7.4%)	1 (2.7%)	0 (0.0%)	3 (2.5%)
Thumb/pencil sucking, *n* (%)	3 (8.8%)	0 (0.0%)	2 (5.4%)	1 (4.5%)	6 (5.0%)
Mouth breathing	9 (26.5%)	16 (59.3%)	23 (62.2%)	2 (9.1%)	50 (41.7%)

*Note:* Continuous data presented as mean ± SD.

^a^ State Anxiety scale of the State‐Trait Anxiety Inventory for children (STAI‐C).

^b^ State Anxiety scale of STAI (STAI Form Y‐1).

After 5 weeks (T1), rehabilitation status (RS) “0” predominated overall (65/120; 54.2%), followed by RS “1” (31/120; 25.8%) and “2” (24/120; 20.0%). At 10 weeks, RS “2” became the dominant category (76/120; 63.3%), while “0” decreased to 17/120 (14.2%) and “1” to 27/120 (22.5%).

**Figure 1:**
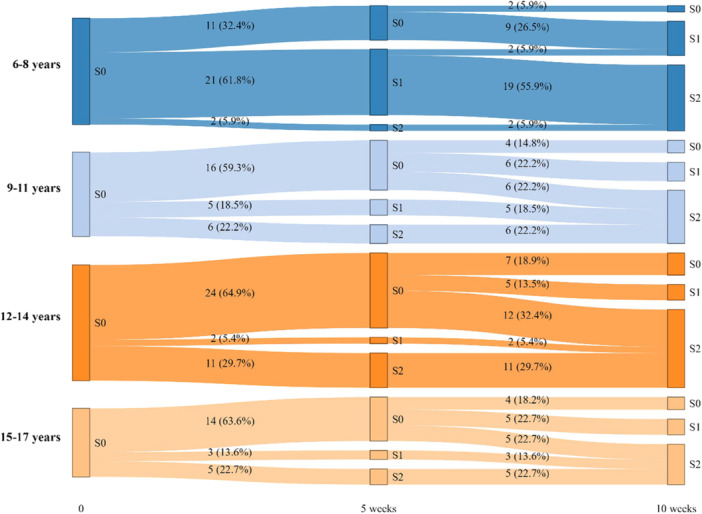
Plots of rehabilitation status transitions between T1 (~5 weeks) and T2 (~10 weeks) in the study cohort (March 2022–June 2024). S*—rehabilitation status (0—pre‐treatment; 1—engrammation; 2—automation).

Age‐stratified patterns differed at 5 weeks (Figure 1, Table 2): the 6–8 years group was mainly at RS “1” (61.8%), whereas no rehabilitation effect was primarily observed in older groups (RS “0”—59%–65%). By 10 weeks, automation (RS “2”) exceeded 59% across all age groups, with the highest proportion in the 12–14 years age group (67.6%).

**Table 2:** Rehabilitation status at 5 and 10 weeks across age groups.

Group	Age	*N*	Rehabilitation status	T1 (5 weeks)	T2 (10 weeks)
1	6–8 years	34	0	11 (32.4%)	2 (5.9%)
			1	21 (61.8%)	11 (32.4%)
			2	2 (5.9%)	21 (61.8%)
2	9–11 years	27	0	16 (59.3%)	4 (14.8%)
			1	5 (18.5%)	6 (22.2%)
			2	6 (22.2%)	17 (63.0%)
3	12–14 years	37	0	24 (64.9%)	7 (18.9%)
			1	2 (5.4%)	5 (13.5%)
			2	11 (29.7%)	25 (67.6%)
4	15–17 years	22	0	14 (63.6%)	4 (18.2%)
			1	3 (13.6%)	5 (22.7%)
			2	5 (22.7%)	13 (59.1%)

### 3.2 Rehabilitation Status Transitions From T1 to T2 and Age‐Dependent Progress

Across the cohort, improvement during the within‐assessment period (5–10 weeks) was observed in 76/120 (63.3%) patients; 44/120 (36.7%) remained unchanged. The magnitude of improvement varied by age (Table 3). Thus, in the 6–8 years group, improvement was most frequent but occurred exclusively as a + 1 RS shift (Δ1: 82.4%; Δ2: 0%). In contrast, 12–14 years showed fewer improvements overall, but the largest proportion of +2 RS shifts (Δ2: 32.4%). The distribution of shift magnitudes (Δ 0/1/2) differed significantly between age groups (Fisher's exact test *p*‐value < 0.001). In addition, a significant within‐group change in RS distribution between 5 and 10 weeks was observed in all age groups (Table 4).

**Table 3:** Rehabilitation status shift (Δ) between 5 and 10 weeks by age group.

Group	Age	*N*	Δ0	Δ1	Δ2
1	6–8 years	34	6 (17.6%)	28 (82.4%)	0 (0.0%)
2	9–11 years	27	11 (40.7%)	9 (33.3%)	7 (25.9%)
3	12–14 years	37	18 (48.6%)	7 (18.9%)	12 (32.4%)
4	15–17 years	22	9 (40.9%)	8 (36.4%)	5 (22.7%)

*Note:* Δ = Rehabilitation status (T2) − Rehabilitation status (T1). Δ0 – Rehabilitation status unchanged (without improvement). Δ1 and Δ2 – Rehabilitation status changed “+1” and “+2”, respectively. Improved = Δ1 + Δ2.

**Table 4:** Paired within‐group change (5 vs. 10 weeks).

Group	Age	*N*	*χ* ^2^	df	*p*‐value
1	6–8 years	34	28.00	2	< 0.001
2	9–11 years	27	14.31	2	< 0.001
3	12–14 years	37	17.72	2	< 0.001
4	15–17 years	22	11.64	2	0.003

*Note: χ* ^2^—Stuart–Maxwell test.

**Figure 2:**
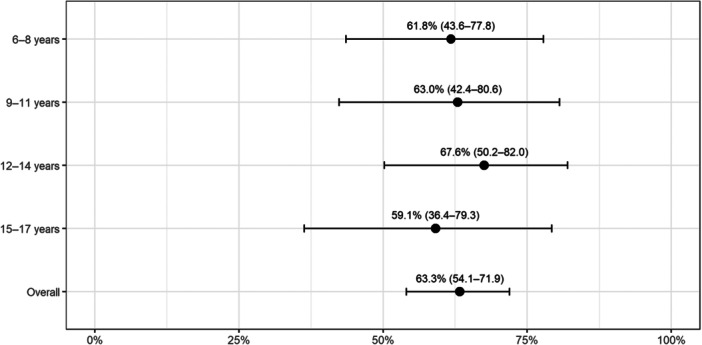
Proportion of patients achieving rehabilitation status 2 (automation) at ~10 weeks in the study cohort (March 2022–June 2024). Points represent proportions; whiskers represent exact 95% binomial confidence intervals.

At 10 weeks (T2), RS 2 (automation) varied across age (Figure 2), with a small effect size (*w* = 0.06; power = 8%).

## 4 Discussion

A consistent improvement in swallowing patterns was observed in a clinical series of children and adolescents with atypical swallowing managed during the wartime period in Ukraine. Between 5 and 10 weeks, the distribution shifted away from the pre‐treatment RS and towards later rehabilitation stages. These findings support the feasibility of a standardized, low‐burden program in real‐world conditions where adherence to conventional, exercise‐heavy myofunctional regimens is often problematic.

In our cohort with atypical swallowing, an increased frequency of specific oral and postural habits is observed; as expected for a clinic‐based referral sample enriched by inclusion criteria, these proportions should not be interpreted as population prevalence. The frequency of mouth breathing, a key risk factor for dentofacial development (Zhao et al. 2021), and lip biting were higher than pooled population‐based estimates reported for children/adolescents (Gyra et al. 2025; Dutta and Verma 2018). The high frequency of mouth breathing in our cohort is consistent with reports that this pattern may occur in children with psychological factors, even without upper airway obstruction (Achmad and Ansar 2021). This aligns with our data, as the 12–14 years subgroup has the highest frequency of this pattern, and mean anxiety scores within the subset (49.7) exceeded a commonly used screening cut‐off (≥ 40) proposed in individual studies for STAI‐S (Dümmler et al. 2025). Under the chronic stress of wartime conditions, such elevated anxiety may reinforce these deleterious oral habits as ingrained compensatory behaviors, requiring a diagnostic approach that accounts for both physiological and psychological stressors. The extremely high frequency of postural habits, such as resting on the hand (up to 100% in those aged 9–11 years), highlights the frequent co‐occurrence of postural behaviors in children referred with myofunctional disorders (Di Vecchio et al. 2019; Quinzi et al. 2020).

This interpretation should be placed in the broader wartime psychosocial context, which is unlikely to be a neutral background for orofacial behaviors. Elevated stress reactions in Ukrainian children and adolescents during the war have been documented, and broader post‐2022 deterioration of child/adolescent mental‐health indicators has also been reported (Martsenkovskyi et al. 2024; McElroy et al. 2024). Furthermore, psychological stress may contribute to the persistence of parafunctional habits (Gyra et al. 2025). Overall, our cohort demonstrates the highest rate of oral/postural habits in the 6–8 and 9–11‐year‐old groups; however, this should be interpreted with caution, as the study included only patients with atypical swallowing. Nevertheless, it stresses the necessity of increased focus on myofunctional therapy to prevent skeletal deformities (Gyra et al. 2025; Homem et al. 2014).

The observed improvement from week 5 to week 10 is consistent with a time‐dependent acquisition process rather than an immediate effect. FroggyMouth is positioned as a low‐burden protocol (15 min/day), intended to facilitate a more “automatic” swallowing pattern without a high‐load exercise schedule (Di Vecchio et al. 2019). From a behavioral perspective, the key advantage of a brief daily routine is adherence (Di Vecchio et al. 2019; Alzoubi et al. 2023); however, our study did not directly measure adherence, so this remains an assumption only.

Across age groups, RS2 at 10 weeks exceeded ~59% in all age groups, with the highest proportion in the 12–14 group (67.6%). The age‐stratified analysis of RS transition magnitudes (Δ0/Δ1/Δ2) suggests heterogeneity in trajectories, but this does not automatically translate into a stable age effect, especially with limited sample sizes. At the same time, the effect size for between‐group differences at 10 weeks was negligible, and the reported power was low. Therefore, we did not detect a clear age‐related difference in short‐term RS2 attainment.

Evidence on FroggyMouth remains limited and heterogeneous. Longer‐term structural/orthodontic changes have been explored in a small prospective cohort treated for 1 year, suggesting potential dental arch modifications beyond short‐term functional staging (Scribante et al. 2024). Additional published evidence includes case‐based series, which support feasibility but do not provide comparative effectiveness estimates (Manzini et al. 2020; Martelli et al. 2024). Another study reported re‐education of swallowing patterns and reaching automatization status in 29 out of 48 patients aged 5–16 years during 10 weeks of treatment (Fellus 2021). Di Vecchio et al. (2019) described the use of FroggyMouth in an orthodontic clinical setting, with follow‐up every 6–8 weeks and a typical treatment duration of ~9 months. Quinzi et al. (2020) enrolled 48 children aged 5–13 years and reported outcomes in 40 completers at 6 months; 82.5% (33/40) achieved correction, and this success was entirely driven by the compliant subgroup. Our endpoint (RS2 at 10 weeks) reflects short‐term attainment of the manufacturer‐defined “automation” stage rather than long‐term stability or orthodontic outcomes, and stabilization with gradual reduction in use over 9–12 months is required (Froggy & Co et al.). Therefore, the direct comparison of the aforementioned studies should be interpreted cautiously, given the lack of long‐term verification of stability/relapse. A shorter time to RS2 in our cohort may also reflect protocol differences, such as incorporating olfaction‐based swallowing automatization and articulation drill exercises (per manufacturer guidance), which may have contributed to earlier improvement (Begnoni et al. 2020). In addition, it has been demonstrated that FroggyMouth is associated with increased lip strength over the course of treatment, and early perioral muscle performance changes (e.g., tone/mimic improvement) may become clinically apparent before complete automation of a new swallowing pattern (Quinzi et al. 2020). On the other hand, nearly half of the patients (51%) were in the phase of active physiological development (ages 6–11), which facilitates rapid and highly predictable outcomes (Di Vecchio et al. 2019). At the same time, the remaining portion of the cohort (49%) consisted of adolescents (ages 12–17), a period during which dental appearance/function and social perception become increasingly relevant (Tekbaş‐Atay and Büyükgöze‐Dindar 2024; Göranson et al. 2023). Although patient compliance was not formally assessed in this study, it can be hypothesized that in both age groups, these developmental factors, combined with proactive parental involvement, may have contributed to achieving the observed results (Nahajowski et al. 2022; Nobre and Pozza 2023).

These clinic‐based data support the practical implementability of a standardized, time‐limited FroggyMouth protocol within routine care pathways. The observed shift toward the protocol‐defined RS2 stage over 10 weeks can be used by clinicians as a short‐term monitoring benchmark in settings where adherence to intensive myofunctional exercises is uncertain.

### 4.1 Limitations

This study has several limitations. The study is an uncontrolled, clinic‐based observational series, which precludes causal inference and limits generalizability. Because a screening log was not prospectively maintained, the numbers assessed for eligibility, excluded, declined participation, and lost to follow‐up are not reported. Swallowing was evaluated clinically and indirectly (saliva swallowing) without instrumental confirmation; intra‐ and inter‐rater agreement were not formally quantified, so misclassification cannot be excluded. Adherence to the daily regimen and adjunct exercises was assessed by parent/guardian report rather than objective monitoring. Follow‐up was limited to 10 weeks; therefore, the stability of the achieved pattern and the risk of relapse beyond this period remain unknown. Different anxiety instruments were used across age groups (STAI‐C in children and STAI‐Y in adolescents); therefore, anxiety results are not directly comparable across the full 6–17‐year range and are presented as age‐stratified descriptive findings only.

## 5 Conclusion

In this clinic‐based observational study of 120 children and adolescents with atypical swallowing, applying the standardized FroggyMouth‐based program demonstrated a shift toward higher rehabilitation status between 5 and 10 weeks, with achievement of the “automation” stage reached by 63.3% (95% CI 54.1–71.9) at 10 weeks. The proportions were broadly comparable across age groups, and no clinically meaningful age‐related differences in short‐term attainment were detected in this sample. Given the uncontrolled design and short follow‐up, the durability of the achieved patterns and the comparative effectiveness of this approach versus other myofunctional approaches remain uncertain; further controlled studies with longer follow‐up are required.

## Author Contributions

Conception and design: Nataliia Makhlynets, Myroslava Kokoshko, and Zinovii Ozhogan. Acquisition of data: Nataliia Makhlynets, Zinovii Ozhogan, and Taras Kotyk. Analysis and interpretation of data: Nataliia Makhlynets, Nataliia Kozan, and Myroslava Kokoshko. Drafting of manuscript: Nataliia Makhlynets and Taras Kotyk. Critical revision: Zinovii Ozhogan and Taras Kotyk. The final version was approved by all authors.

## Funding

The authors have nothing to report.

## Ethics Statement

The study was conducted in accordance with the principles of the Declaration of Helsinki and was approved by the Ethics Committee of the Ivano‐Frankivsk National Medical University.

## Consent

Written and verbal informed consent was obtained from all children's parents before participating in the study.

## Conflicts of Interest

The authors declare no conflicts of interest.

## Data Availability

Data supporting findings are available from the corresponding author upon reasonable request.
